# More Choosy for Minor Foods: Diet, Seasonality and Food Selection in Sympatric Frugivorous and Folivorous Lemurs

**DOI:** 10.1002/ece3.71069

**Published:** 2025-03-13

**Authors:** Mitchell T. Irwin, Vololonirina Rahalinarivo, Bruno Ramorasata, Jean‐Luc Raharison, Jean‐Freddy Ranaivoarisoa, Chloé N. M. Gherardi, Jessica M. Rothman

**Affiliations:** ^1^ Department of Anthropology Northern Illinois University DeKalb Illinois USA; ^2^ NGO Sadabe Antananarivo Madagascar; ^3^ Mention Anthropobiologie et Développement Durable, Université d'Antananarivo Antananarivo Madagascar; ^4^ Department of Biological Sciences Northern Illinois University DeKalb Illinois USA; ^5^ Department of Anthropology Hunter College New York New York USA; ^6^ Graduate Center City University of New York New York New York USA

**Keywords:** diet, *Eulemur fulvus*, Madagascar, nutrition, *Propithecus diadema*, season, Tsinjoarivo

## Abstract

Despite traditional dietary categories common in primatology (folivore, frugivore, insectivore), most primates use one or more food types beyond their primary one. Among lemurs, folivores tend to have a complicated, long gastrointestinal tract with an enlarged cecum, shearing teeth, and microbiome adaptations to foliage, while frugivores have simple teeth and guts and fast gut passage. Yet frugivores often eat some leaves, and folivores eat some fruit, and little is known about the selection rules they follow within each food type. We quantified diet and food chemistry for two sympatric rainforest lemurs: a cathemeral frugivore (brown lemur, *Eulemur fulvus* ) and a diurnal folivore (diademed sifaka, *Propithecus diadema* ) over 18 and 13 months. Brown lemurs ate 78.3% fruit and 13.0% leaves; sifakas ate 52.8% leaves and 37.9% fruit/seed; both ate fruit/seed most in the resource‐abundant season, increasing leaf/flower consumption in the lean season. Both had diverse diets (128 and 81 plant species) but selected almost entirely different species; however, within fruit and leaf categories, their foods overlapped substantially in nutritional content. They were more selective in their secondary foods: sifakas selected higher‐energy fruits and brown lemurs selected leaves higher in protein and minerals. This indicates a balancing function: frugivores selecting leaves strategically to compensate for low‐protein, low‐mineral fruit and folivores selecting fruit/seed to compensate for low‐energy leaves. That said, it is puzzling why sifakas ignored some leaf species eaten by brown lemurs that were high in protein and minerals—this suggests these nutrients are not prioritized or limiting for sifakas. Other factors likely contribute to the mutual exclusivity in food selection, particularly plant secondary metabolites not measured here or the (poorly‐understood) metabolic costs of eating nutrients in excess. More research is needed to fully understand food choices, how these promote niche differentiation, and their consequences for animals, communities, and ecosystems.

## Graphical abstract

Primates are deliberate and intelligent foragers and are presumed to select foods that maximize their fitness, yet little is known about the actual rules of food selection based on the nutritional chemistry of those foods, and less is known about how sympatric primates differ. We report striking divergence in food selection for two sympatric lemurs (one mainly frugivorous but consuming some leaves, one mainly folivorous but consuming some fruit). Each was more selective for its secondary food, suggesting a strategic balancing use of those foods in the context of the broader diet.

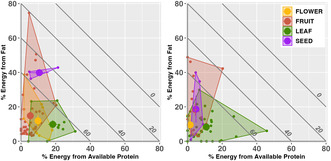

## 1 Introduction

Diets across the Primate order are extremely varied, and primatologists have found it useful to divide species, on a first pass, according to their primary (most utilized) food (Hawes et al. 2024). This mainly yields frugivores, folivores, exudativores, and faunivores, with the additional term “omnivore” used when diets span multiple trophic levels. These categories are useful in understanding broad divergences in anatomical and physiological adaptation to foods, but also obscure considerable variation; almost all primates are not fully committed to one category. Many fall roughly evenly between two, including frugi‐folivores such as *Alouatta* (Righini et al. 2017), *Propithecus* (Irwin et al. 2014) and western *Gorilla* (Remis 1997), or insectivore‐frugivores such as *Saimiri* (Lima and Ferrari 2003), and some have substantial contributions from three food types, such as *Cercopithecus* (fruit, leaves, and insects: Bryer et al. 2015). Although our understanding of diet mixing is incomplete, it seems clear that folivores can benefit from adding some energy‐rich fruit or seeds to offset the slowly digestible, low‐energy leaves (Rothman et al. 2011). Similarly, frugivores often get little protein from fruits and benefit from supplementing with protein‐rich leaves or insects (Rothman et al. 2014). In addition to this mixing of food types, primates show considerable dietary plasticity, often resulting in high diversity of species exploited; they can include more than 100 species across years/lifetimes and dozens of species per day (Chaves et al. 2023).

It is thought that primates use these complex diets to optimize the balance between nutritional payoffs and various costs: energy expenditure (including movement), ingestion of detrimental chemicals, exposure to predators, and interspecific competitors; in other words, primates choose specific foods and quantities to optimize fitness, thereby ignoring other potential foods in the environment, which may be consumed by other species (Chapman et al. 2012). Much of this interspecific divergence reflects evolved intrinsic differences in physiology, such as dental anatomy, salivary and gastrointestinal digestive enzymes, gut passage time, gastrointestinal tract morphology and function, gut microbiome, and capacity for neutralizing plant secondary metabolites (PSMs) (Lambert and Rothman 2015). Although basic nutrient acquisition goals are shared, species' differing intrinsic adaptations mean that they differ in which foods are chemically ideal for meeting those goals. This divergence can be seen in sympatric primates, including close relatives such as congeners (Overdorff 1993; Justa et al. 2019), and species pairs with the same primary diet type (Campera et al. 2021; Wright et al. 2011), which all show dietary divergence at least during parts of the year. Considerable research has been devoted to niche separation (primarily along spatial, temporal and chemical axes) and community assembly in sympatric primates and other taxa, both as a window on past evolutionary processes and to better understand ecosystem assembly and the specifics of coexistence for species in the present (Ganzhorn 1989; Warren et al. 2008).

Despite considerable research documenting primate diets and dietary divergence in the wild, few studies have collected the nutrition data needed to understand the chemical factors governing primate food selection, and thus, these remain obscure (Felton, Felton, Lindenmayer, and Foley 2009; Felton and Lambert 2024). These studies generally set out to compare the nutritional composition of foods and non‐foods or seek correlations between nutritional values and preference. Results have been inconsistent: many nutrients affect foraging choices in some species but not others, and some studies find little or no significant relationships in the variables tested (Chapman and Chapman 2002). Primates do sometimes select high‐protein foods (Milton 1979; Ganzhorn 2002; Calvert 1985), but about half of the studies testing for protein selection fail to find it, possibly because it only occurs in more nitrogen‐limited environments (Ganzhorn et al. 2017). Protein is known to be critical for growth and reproduction and may limit many animal populations (White 1993), but this may not apply universally across primates for several reasons. Primates grow slowly, produce dilute milk, and may therefore have a lessened need for protein. Primate folivores in particular consume high‐protein foods (leaves) and therefore may not need to select high‐protein species (Oftedal 1991), except in habitats with leaves unusually low in protein (Ganzhorn et al. 2017). In situations where protein isn't the limiting factor, primates may prioritize non‐protein energy (lipids and carbohydrates) while consuming surplus protein (Rothman et al. 2011) or follow different food selection rules entirely. More recently, researchers acknowledge that nutritional strategies vary across species and that animals may sometimes seek a specific nutrient balance rather than maximizing or optimizing the intake of one nutrient (Robbins et al. 2007; Raubenheimer et al. 2015; Felton, Felton, Raubenheimer et al. 2009).

Finally, some studies have suggested an important role for minerals in food selection. Some African studies have suggested that certain foods are targeted for sodium, reflecting the generally low sodium levels in foods (Rothman et al. 2006). Lemur diets are often low in calcium (Irwin et al. 2017), suggesting that some foods are consumed for this micronutrient; bamboo lemurs may target high‐sulfur foods due to their need for sulfur in cyanide detoxification (Eppley et al. 2017). However, due to the relatively low requirements for minerals, it seems likely that macronutrient needs may dominate feeding choices, with micronutrient needs met by targeting a smaller list of key foods.

To date, few studies have quantified dietary divergence and overlap in sympatric lemurs across seasons. Overdorff (1993) reported that the two frugivores *Eulemur rufifrons* and *E. rubriventer* at Ranomafana showed considerable dietary convergence in some months, but broader differences in the use of unripe fruit and mature leaves, and diverging lean season strategies. Follow‐up studies adding *Varecia variegata* and *Propithecus edwardsi* (a frugivore and a folivore) showed relatively little dietary overlap, with the folivore having the highest dietary diversity (Erhart et al. 2018; Wright et al. 2011). Two folivores ( *Avahi meridionalis* and *Lepilemur fleuretae* ) showed very limited dietary overlap in resource‐rich months and moderate overlap in resource‐poor months (Campera et al. 2021), and *Microcebus* species' diets diverged strongly in species pairs at Ankarafantsika (Thorén et al. 2011) and Kirindy (Dammhahn and Kappeler 2008). However, divergence is not the rule: at Berenty, *Lemur catta* and *Propithecus verreauxi* , two species with very different physiological adaptations, often share the same top food, *Tamarindus indica* (Jolly 1966; Simmen et al. 2003), and three cheirogaleid lemurs at Mandena showed extensive dietary overlap (Lahann 2007).

Even fewer studies have taken the extra step of comparing the nutritional chemistry of sympatric species' foods across seasons. Ganzhorn (1988) found that seven lemur species at Analamazaotra consumed largely different species of fruit and leaves; the leaves differed among consumers, largely in extractable protein, hemicellulose, alkaloids, and condensed tannins, while the fruits differed in condensed tannins. Two additional folivores sampled at Ampijoroa also differed in the chemistry of selected leaves. Yamashita (2008) documented dietary divergence of *L. catta* and *P. verreauxi* at Beza Mahafaly, with the latter consuming more protein and fewer sugars. Campera et al. (2021) documented strong dietary divergence in the sympatric folivores *Avahi meridionalis* and *Lepilemur fleuretae* , and some nutritional divergence, with the former showing seasonal preference for high‐protein and low‐polyphenol foods. We therefore have very little knowledge of how much sympatric lemurs differ in diet and food selection rules, including targeted comparisons within food types (e.g., whether folivores select leaves similar to those eaten by frugivores). This limits our understanding of past niche divergence and current community structure and habitat needs.

Here, we report diet and food nutritional characteristics of two conspecific lemurs with considerable dietary divergence: one diurnal folivore (diademed sifaka, *Propithecus diadema* ) and one cathemeral frugivore (brown lemur, *Eulemur fulvus* ). We explore the following questions. First, how much do their diets overlap in terms of plant parts (how much does the folivore eat fruit, and the frugivore eat leaves)? Second, how do they compare in species diversity in the diet? We predict higher dietary diversity in *P. diadema* due to its emphasis on leaves, which more often contain PSMs (Marsh et al. 2006; Hending et al. 2024; Erhart et al. 2018). Third, how much do their diets overlap in terms of plant species? Fourth, to the extent that they select different species, do their selections differ chemically—in nutrients and tannins (plant defensive compounds that prevent protein absorption)? We predict that *E. fulvus* will select higher‐protein leaves (to offset a low‐protein fruit diet), and *P. diadema* will select more energy‐rich fruits and seeds (to offset a low‐energy leaf diet), and we also expect stronger selection for mineral content in *E. fulvus* , as fruits tend to be lower in minerals than leaves (Irwin et al. 2017).

## 2 Methods

This research complied with protocols approved by the Northern Illinois University IACUC, adhered to the American Society of Primatologists Principles for the Ethical Treatment of Non‐Human Primates and Code of Best Practices for Field Primatology, and adhered to the legal requirements of Madagascar.

### 2.1 Study Site and Subjects

This study was conducted at the Ankadivory forest (19°42′59″ S, 47°49′18″ E, 1320–1550 m elevation) within the Tsinjoarivo‐Ambalaomby New Protected Area (25,687 ha). Roughly half of the protected area is forested; Ankadivory contains relatively undisturbed primary rainforest with human settlement nearby and has experienced limited tree extraction and disturbance. The site is just north of the Onive river and part of the eastern rainforest corridor atop Madagascar's eastern escarpment, which divides the central plateau from the eastern lowlands (Irwin et al. 2019; Rakotomalala et al. 2017). Rainfall at Vatateza camp (4 km to the east) averages 2632 mm, of which 1697 mm (64%) falls during December–March (Irwin, unpublished). We refer to the hotter, rainy season (November to April) as the “abundant season” (Irwin et al. 2015) because of the higher availability of flowers, fruit, and young leaves compared to the “lean season” (May to October). Annual average temperature during the study period was 15.9°C; monthly averages ranged from 11.3°C (July 2016) to 18.8°C (February 2017).

The study species are the largest lemurs at Tsinjoarivo: *Eulemur fulvus* (adult mass at Ankadivory 2222 ± 241 g; Irwin unpublished data) lives in multi‐male, multi‐female social groups ranging from 3 to 12 individuals, while *P. diadema* (adult mass 4993 ± 338 g; Irwin et al. 2019) lives in small groups with one adult male and 1–2 adult females. For both, Tsinjoarivo represents the southern tip and the highest elevation site of their geographic range.

### 2.2 Study Groups and Behavioral Data Collection

We sampled one group of each species. *Eulemur fulvus* group ANKA1 had 6 individuals at the beginning of the study (1 adult female, 4 adult males, 1 subadult) but in October 2016, the adult female died and there was substantial turnover (immigration and emigration). Beginning in November 2016, the group in the same home range had 11 individuals (4 adult females, 4 adult males, 3 immatures), excluding dependent infants. A second demographic change occurred in February 2017: three collared adult males and one uncollared individual emigrated, and one new adult male arrived. For each data collection cycle, we focused on six focal animals, all recognizable using collars and/or the lack of collars (process of elimination). The dataset includes 16 individuals (5 adult females, 9 adult males, and 2 subadults). *P. diadema* group CONT4 had five individuals at the beginning of the study (one adult female, one adult male, one 3‐year‐old female, one 1‐year‐old female offspring, and a female infant) and experienced the birth of two female infants in June 2017; we collected data on the four oldest individuals. These two study groups overlapped in home range (the smaller *P. diadema* home range of ~81 ha was within the larger *E. fulvus* home range of ~135 ha).

As part of a long‐term study, we captured selected individuals (details in Irwin et al. 2019) with a veterinarian to ensure animal safety. One adult female per group was fitted with a radio collar (Advanced Telemetry Systems, Isanti, MN, USA); other animals were given olefin collars with aluminum tags, with reflective printed collars and tape for *E. fulvus* .

We collected data from July 2016 to December 2017 for *E. fulvus* (18 months, one 2‐week cycle per month, 6 days per week, 2 focal animals per day) and from July 2016 to July 2017 for *P. diadema* (13 months, one 1‐week cycle per month, 4 focal animals per day). For *E. fulvus* , we collected data during midnight‐to‐midnight “days”, with three groups of research technicians working shifts of roughly 8 h each; for *P. diadema* , we collected data from roughly dawn to dusk. We recorded feeding data continuously (Altmann 1974), noting start and stop time, food species and plant part consumed for each feeding bout. In total, the *E. fulvus* dataset comprised 18 monthly cycles, 434 days (of which 413 were complete days with < 8% “out of sight” time), 10,362 observation hours and 1622 feeding hours. The *P. diadema* dataset comprised 13 monthly cycles, 308 days (of which 267 were complete days with animals found in morning sleep trees and < 8% “out of sight” time), 3149 observation hours and 864 feeding hours.

### 2.3 Food Sample Collection and Lab Analysis

We collected representative samples of each species' foods using tree climbing and/or pruning poles immediately following data collection cycles. Samples were processed to reflect both ingestion and digestion: plant parts that were dropped before ingestion or passed undigested in feces were removed before drying. We also sampled leaves of 11 species never seen to be consumed by either species, five at Ankadivory in July 2017 and six at Mahatsinjo in June–July 2019 (7.5 km NW of Ankadivory). Samples were dried for three to 5 days in a Nesco electric dehydrator at 41°C, then placed in a ziplock bag with desiccant and stored in the dark after weight had stabilized.

We analyzed samples for macronutrient content at Hunter College or Northern Illinois University using identical techniques. First, samples were milled to a uniform 1‐mm particle size using a Thomas Wiley MiniMill. Chemical analysis followed standard techniques (Rothman et al. 2012), summarized briefly here. Tannins were assayed using the acid butanol assay, which produced a pink‐red color in the presence of tannins; samples with measured absorbance > 0.1 were considered “tannin‐positive”. Crude protein was determined via combustion (Leco FP828P), multiplying the nitrogen content by 6.25 to estimate protein, and available protein was determined using the “AvailN” method (DeGabriel et al. 2008), which simulates digestion. All samples were run without polyethylene glycol (PEG, a tannin‐deactivating agent), but for samples that screened positive for tannins, we also ran the assay with PEG added. Crude fat was determined using ether extract. Water‐soluble carbohydrates (WSC) were extracted with boiling water and quantified using the phenol‐sulfuric acid assay, with sucrose as a standard. All three fractions of fiber (NDF, ADF and lignin) were assayed using the sequential neutral detergent fiber (NDF) technique (Van Soest et al. 1991) in an A200 Fiber Analyzer (ANKOM, Macedon, NY); NDF assays used alpha‐amylase but not sodium sulfite. Dry matter content was determined by drying a subsample at 105°C for 24 h to remove moisture, and ash content was determined by heating at 550°C for 3 h. All nutrient contents are reported on a dry matter basis.

Mineral analyses were performed at Dairy One Forage Lab (Ithaca, NY) and assayed for Ca, P, Mg, K, Na, Fe, Zn, Cu, and Mn. Samples were extracted with an acid solution (HNO_3_+ HCl) then hydrogen peroxide, and analyzed using a Thermo iCAP Pro XP Inductively Coupled Plasma Radial Spectrometer.

Total non‐structural carbohydrates (TNC) were estimated as:

$$\mathrm{TNC}=100-\left(\mathrm{Ash}+\text{Crude}\;\mathrm{Fat}+\mathrm{NDF}+\text{Available Protein}\left(\text{with}\;\mathrm{PEG}\right)\right)$$

Crude protein should not be used in this subtraction as fiber‐bound protein would be double‐counted in crude protein and NDF. We therefore used “available protein with PEG” (i.e., with tannins deactivated) as this is the closest equivalent to the “crude protein minus acid detergent indigestible crude protein” used in previous studies (it is lower than crude protein as it excludes fiber‐bound nitrogen, but not as low as “Available Protein without PEG”, which further excludes tannin‐bound protein). When samples screened negative for tannins, we used “Available Protein without PEG” instead.

We estimated energy as:

$$\begin{array}{cc} \text{Energy}\;\left(\mathrm{kJ}/g\right)\;=\; & \left(\mathrm{TNC}\times 16.736\right)+\left(\mathrm{NDF}\times 12.552\times {\mathrm{DC}}_{\mathrm{NDF}}\right)\\ & +\left(\text{Adjusted}\;\mathrm{fat}\times 37.656\right)+\left(\text{Available Protein}\times 16.736\right) \end{array}$$

where nutritional variables are expressed as proportions, adjusted fat equals crude fat for non‐leaves but equals “crude fat – 1” for leaves, and the digestibility coefficients (DC_NDF_) are 0.28 for *E. fulvus* and 0.43 for *P. diadema* (Cassalett 2022).

### 2.4 Data Analysis

We expressed diet composition in terms of feeding time (i.e., the amount of feeding devoted to one food type as a percentage of total feeding time). When describing diet in terms of plant part, we used weighted averages so that each calendar month's diet was given even representation, whether it was sampled once or twice. Water accounted for 0.33% of “feeding” records for *E. fulvus* but was excluded from analyses.

We calculated dietary diversity for each lemur species across the whole dataset and within monthly cycles, using two measures: species richness and Simpson's D, a diversity index that reflects both the species diversity and the evenness of feeding across those species, defined as:

$$D=\frac{1}{\sum_{i=1}^{S}p_{i}^{2}}$$

where *S* = the total number of food species represented, and *p*_i_ = the proportion of feeding time represented by species *i* (animal species were included). We tested for correlations between monthly diversity measures and folivory using Spearman's correlations and for lean versus abundant season differences using Wilcoxon rank sum tests (*α* = 0.05).

Because our feeding databases are large, several food species were recorded for very little feeding time (and could represent misidentifications). These are included in overall species lists (Tables S1 and S2), but for the analyses comparing the lemurs' “selected foods”, we used thresholds to exclude rarely‐used foods. We produced three datasets for analysis. For the first, “all foods” (Table S3), we considered any food (plant part & species combination) that represented > 0.1% of feeding time (*n* = 66 sampled foods for *E. fulvus* , *n* = 58 for *P. diadema* ). The second, “fruits + seeds” (Table S4) included all fruits and seeds in the first database ( *E. fulvus* : *n* = 43; *P. diadema* : *n* = 24, of which 8 were shared). For the third, “leaves” (Table S5), we expanded sampling to consider any leaf representing > 0.2% of leaf feeding time ( *E. fulvus* : *n* = 16, of which 15 were sampled nutritionally; *P. diadema* : *n* = 38, of which 31 were sampled nutritionally; 6 species were on both lists), plus 11 non‐foods. This allowed a greater investigation of leaves selected by *E. fulvus* , only represented by 11 in the first database.

Our data structure has a limited ability to correct for type 1 error (false discovery rate), as we were limited by the number of leaf or fruit species eaten by each lemur, and we generated a large number of nutritional variables (19). Rather than explicitly attempting to adjust p‐values, we note the number of expected type 1 errors in each series of univariate analyses. We used Wilcoxon rank sum tests for three pairwise comparisons: *E. fulvus* foods vs. *P. diadema* foods, *E. fulvus* foods vs. non‐foods (the latter included *P. diadema* foods), and *P. diadema* foods vs. non‐foods (the latter included *E. fulvus* foods). We also used Spearman's correlations to explore the relationship between importance in the diet (% of feeding time represented by a food species) and nutritional variables. We used Right‐Angled Mixture Triangles (Raubenheimer 2024) for the “all foods” database to visualize the balance between macronutrients in foods, variation across food types and consumers, and Principal Components Analysis to visualize overlap between species in leaves and fruits/seeds separately across all macronutrients and all mineral variables. For pairs of variables with a correlation coefficient > 0.90 or < −0.90 (Tables S6–S8), we excluded one of the pair from the Principal Components Analysis. Statistical analyses were conducted using R 4.3.2 (R Core Team 2024).

## 3 Results

### 3.1 Plant Parts Consumed and Seasonality

**FIGURE 1:**
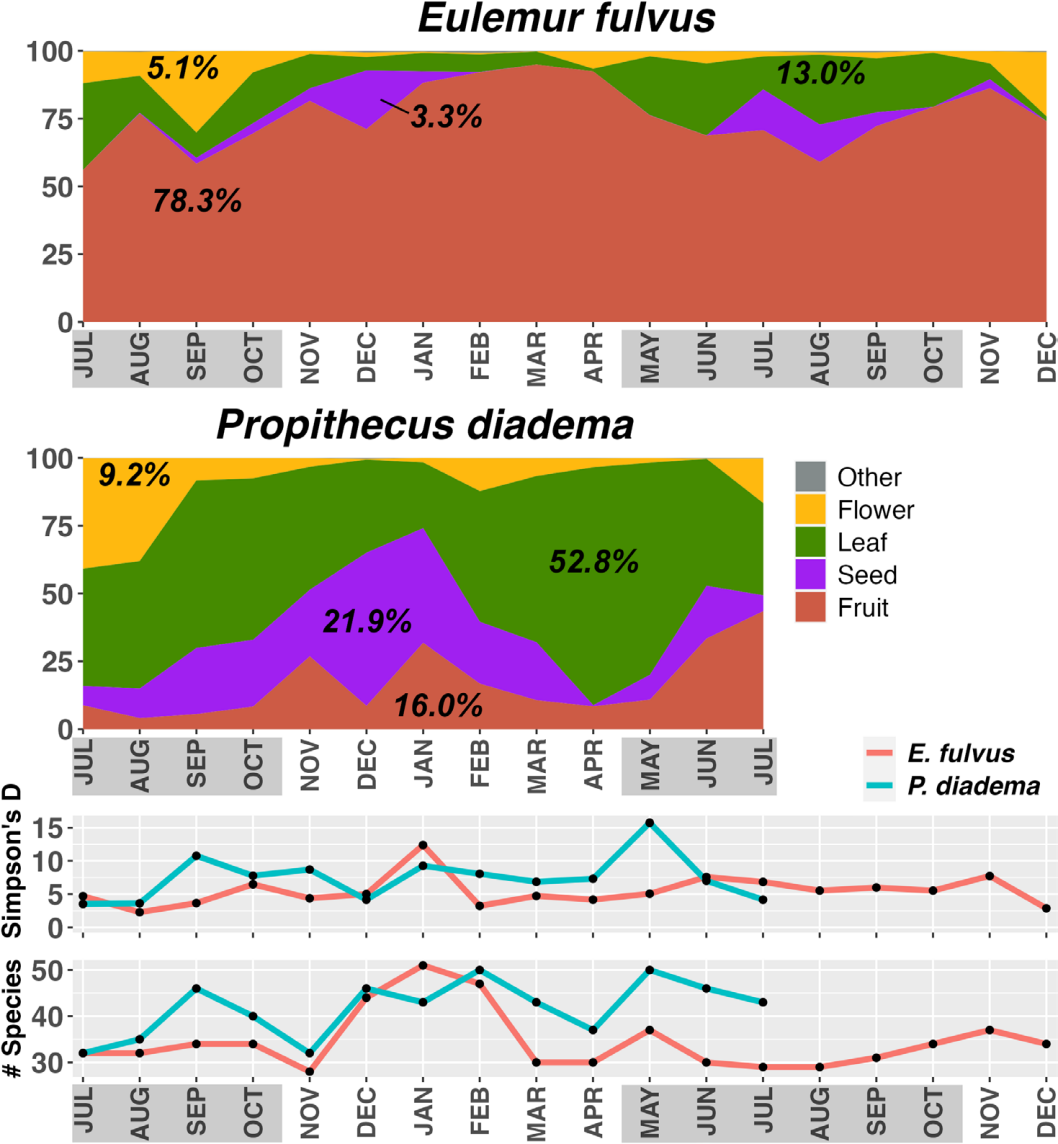
Composition of the diet (percentage of feeding time devoted to fruit, seed, leaf, flower and other foods, from continuous data collection, top) and monthly dietary diversity (bottom) for *Eulemur fulvus* (July 2016–December 2017) and *Propithecus diadema* (July 2016–July 2017) at Ankadivory, Tsinjoarivo. Average diet composition values are weighted averages, with each calendar month given equal weighting in the value; lean season months are highlighted in gray.

For *Eulemur fulvus* , fruit was the top item (weighted average feeding time across 18 months: 78.3%; Figure 1), followed by leaves (13.0%), flower parts (including fleshy peduncles, 5.1%), seeds (3.3%), animals (0.11%), exudate (0.089%), soil (0.064%), galls (0.014%), rotten wood (0.0048%), and fungus (0.0012%). For *Propithecus diadema* , foliage was the top item (weighted average across 13 months: 52.8%), followed by seeds (21.9%), fruit (16.0%), flower parts (9.2%), soil (0.13%) and galls (0.0071%). Both had similar seasonal shifts, with fruit/seed consumption highest in abundant season months (peaking in December–January for *P. diadema* and December–April for *E. fulvus* ). Lean season months saw increased consumption of leaves and/or flowers, but the leaf/flower balance varied among months and across years. The 2017 lean season for *P. diadema* was unusual in that there was a large crop of fruit which was highly consumed (*Syzygium parkeri*). Despite these similarities, the baselines set the species apart: *E. fulvus* always had higher fruit consumption (56%–95% of monthly feeding) than *P. diadema* (4%–44%), and *P. diadema* had higher leaf consumption (24%–87% vs. 1%–32%).

### 3.2 Dietary Diversity, Species Consumed and Dietary Overlap

For the full datasets, Simpson's Index of Dietary Diversity was similar for the species: 16.41 for *P. diadema* and 15.25 for *E. fulvus* ; monthly indices were higher for *P. diadema* in 8 of 13 months (Figure 1). *E. fulvus* consumed 128 plant species, plus invertebrate and vertebrate animals (chameleons, frogs, spiders and unidentified “insects”); plant foods belonged to 50 plant families, dominated by Rubiaceae (Tables 1 and 2). *P. diadema* consumed 81 plant species and no animal matter; plant foods belonged to 38 families, dominated by Loranthaceae and Clusiaceae. Forty‐three species were consumed by both lemurs. For both diversity variables (Simpson's Index and species richness) and both species, there were no significant correlations between diversity in a month and leaf consumption, and no significant difference between lean and abundant season months.

**TABLE 1:** Top 10 consumed plant species for *Eulemur fulvus* and *Propithecus diadema* at Ankadivory, Tsinjoarivo (based on 10,362 h of focal animal observation across 18 months and 3149 h across 13 months, respectively).

Vernacular name	Parts consumed	Scientific name	% Feeding time
*Eulemur fulvus*			
Valotra	RFSD, URFSD	Rubiaceae: *Antirhea borbonica*	20.75
Tavolo maladia	RFSD, URFSD	Lauraceae: *Cryptocarya rigidifolia*	5.64
Nonoka	RFSD, URFSD	Moraceae: *Ficus antandronarum*	5.32
Afitsokina	URFSD, RFSD	Malvaceae: cf. *Grewia*	4.54
Rotra fotsy	RFSD, URFSD, BD/FL, YL	Myrtaceae: *Syzygium parkeri*	4.18
Tavolo maladia 2	RFSD, URFSD	Lauraceae: *Cryptocarya* sp.	3.94
Fatsikahitra tenany	RFSD, URFSD, BD/FL	Rubiaceae: *Coptosperma* sp.	3.84
Voanananala	RFSD	Rubiaceae: cf. *Chassalia*	2.95
Velatra	YL, ML	Acanthaceae: *Acanthopale madagascariensis*	2.94
Ramiavotoloho	YL, URFSD, RFSD, BD/FL	Annonaceae: *Ambavia* cf. *capuronii*	2.88
*Propithecus diadema*			
Tongoalahy SL	BD/FL, YL, URFSD, RFSD	Loranthaceae: *Bakerella clavata* var. 2	13.73
Maintipototra	YL, SD, URFSD, RFSD	Sapindaceae: *Doratoxylon* sp.	9.92
Kimbaletaka	SD, YL, RFSD	Clusiaceae: *Garcinia tsaratananensis*	7.08
Tongoalahy BL	YL, URFSD, RFSD, BD/FL	Loranthaceae: *Bakerella clavata* var. 1	7.08
Vahimainty	YL, URFSD	Apocynaceae: cf. *Plectaneia*	6.75
Voamalambotaholahy	YL, SD	Clusiaceae: *Garcinia* sp.	6.24
Maimbovitsika	YL, BD/FL, URFSD, SD, RFSD	Pittosporaceae: *Pittosporum verticillatum*	5.11
Kimba tenany	YL, SD, URFSD	Clusiaceae: *Symphonia microphylla*	4.83
Tsiramiramy	SD, YL, URFSD	Anacardiaceae: *Abrahamia ditimena*	4.76
Rotra fotsy	RFSD, URFSD	Myrtaceae: *Syzygium parkeri*	4.60

*Note:* Feeding time on water was excluded, but percentage totals cover all feeding time, including rotting wood and non‐plant foods (soil, animal matter). For each species, the plant parts consumed are listed in decreasing order of importance in the diet, with very minor foods (< 500 s) excluded.

Abbreviations: BD/FL, flower buds and flowers; ML, mature leaves; RFSD, ripe fruit with seed; SD, seed; URFSD, unripe fruit with seed; YL, young leaves.

**TABLE 2:** Top five plant families represented in the diet of *Eulemur fulvus* and *Propithecus diadema* at Ankadivory, Tsinjoarivo (based on 10,362 h of focal animal observation across 18 months and 3149 h across 13 months, respectively).

Family	% Feeding time/Number of species
*Eulemur fulvus*	
Rubiaceae	35.72 (19)
Lauraceae	11.26 (6)
Malvaceae	9.23 (6)
Moraceae	8.93 (4)
Myrtaceae	4.23 (7)
*Propithecus diadema*	
Loranthaceae	20.93 (3)
Clusiaceae	18.45 (5)
Sapindaceae	12.21 (3)
Apocynaceae	7.29 (2)
Myrtaceae	6.29 (7)

*Note:* Expressed as a percentage of all feeding time, including non‐plant foods.

Dietary overlap in species consumed varied across months. Monthly feeding time spent on species also eaten by the other lemur was 20.9% ± 19.8% SD (range 0.4–69.0) for *E. fulvus* and 41.63% ± 26.6% (range 2.3–83.4) for *P. diadema* ; overlap peaked in December 2016 (83.4% and 69.0%). However, these numbers include species for which the lemurs ate different plant parts, and capture overlap where a food is highly consumed by one lemur but accounted for minimal feeding for the other. Inspection of food lists reveals little meaningful overlap between the lemurs: for the “all foods” database (> 0.1% of feeding time), only 10 foods of the 114 tested were present for both species (7 fruits, 2 leaves, 1 seed). Further, only four species accounted for more than 1% of feeding time for both lemurs. Two of these, *Syzygium parkeri* (4.18% and 4.60% of feeding time for *E. fulvus* and *P. diadema* ) and *Abrahamia ditimena* (1.77% and 4.76%), were treated differently by the two species: *E. fulvus* swallowed both whole and passed seeds undigested, while *P. diadema* masticated the whole fruit with seed for *S. parkeri* and mostly consumed the seed while discarding the flesh for *A. ditimena*. *Garcinia tsaratanensis* contributed 7.08% of *P. diadema* feeding and 1.06% of *E. fulvus* feeding (> 90% of which was on seeds for both species). Finally, *Symphonia* sp. (Kimba tenany) contributed 1.26% of *E. fulvus* feeding and 4.83% of *P. diadema* feeding (almost all flower buds for *E. fulvus* and young leaves for *P. diadema* ). Though each species had a long list of leaves consumed, 51 and 74 species for *E. fulvus* and *P. diadema* , respectively, overlap was minimal (Table 3; Tables S1, S2 and S5); only two contributed to > 0.2% of leaf‐feeding on both lists (*Tournefortia puberula* contributed 2.30% of *E. fulvus* leaf‐feeding and 2.35% of *P. diadema* leaf‐feeding; *Oncostemum* cf. *acuminatum* contributed 2.01% and 0.21%, respectively).

**TABLE 3:** Top 10 consumed leaf species for *Eulemur fulvus* and *Propithecus diadema* at Ankadivory, Tsinjoarivo (based on 10,362 h of focal animal observation across 18 months and 3149 h across 13 months, respectively).

Vernacular name	Scientific name	% Leaf feeding time
*Eulemur fulvus*		
Velatra	Acanthaceae: *Acanthopale madagascariensis*	20.51
Malambovony kelifofona	Erythroxylaceae: *Erythroxylum nitidulum*	19.20
Ramiavotoloho	Annonaceae: *Ambavia* cf. *capuronii*	18.44
Vahiramy	Cucurbitaceae: *Raphidiocystis* sp.	17.73
Andrarezina	Cannabaceae: *Trema orientalis*	8.46
Anambolotsangana	Acanthaceae: *Isoglossa gracillima*	4.30
Fiatsinakoho 2	Heliotropiaceae: *Tournefortia puberula*	2.30
Kalafambakaka	Primulaceae: *Oncostemum* cf. *acuminatum*	2.01
Apana	Moraceae: cf. *Ficus* sp.	1.66
Vahimaintilany	Fabaceae: *Abrus precatorius*	1.09
*Propithecus diadema*		
Vahimainty	Apocynaceae: cf. *Plectaneia*	13.01
Voamalambotaholahy	Clusiaceae: *Garcinia* sp.	11.96
Tongoalahy SL	Loranthaceae: *Bakerella clavata* var. 2	11.28
Kimba tenany	Clusiaceae: *Symphonia microphylla*	9.22
Maintipototra	Sapindaceae: *Doratoxylon* sp.	9.01
Tongoalahy BL	Loranthaceae: *Bakerella clavata* var. 1	8.23
Maimbovitsika	Pittosporaceae: *Pittosporum verticillatum*	8.01
Rafy	Primulaceae: *Maesa lanceolata*	4.54
Tsilaitra	Hamamelidaceae: *Dicoryphe* sp.	2.49
Fiatsinakoho 2	Heliotropiaceae: *Tournefortia puberula*	2.35

### 3.3 Nutritional Comparisons Across and Within Food Types

**FIGURE 2:**
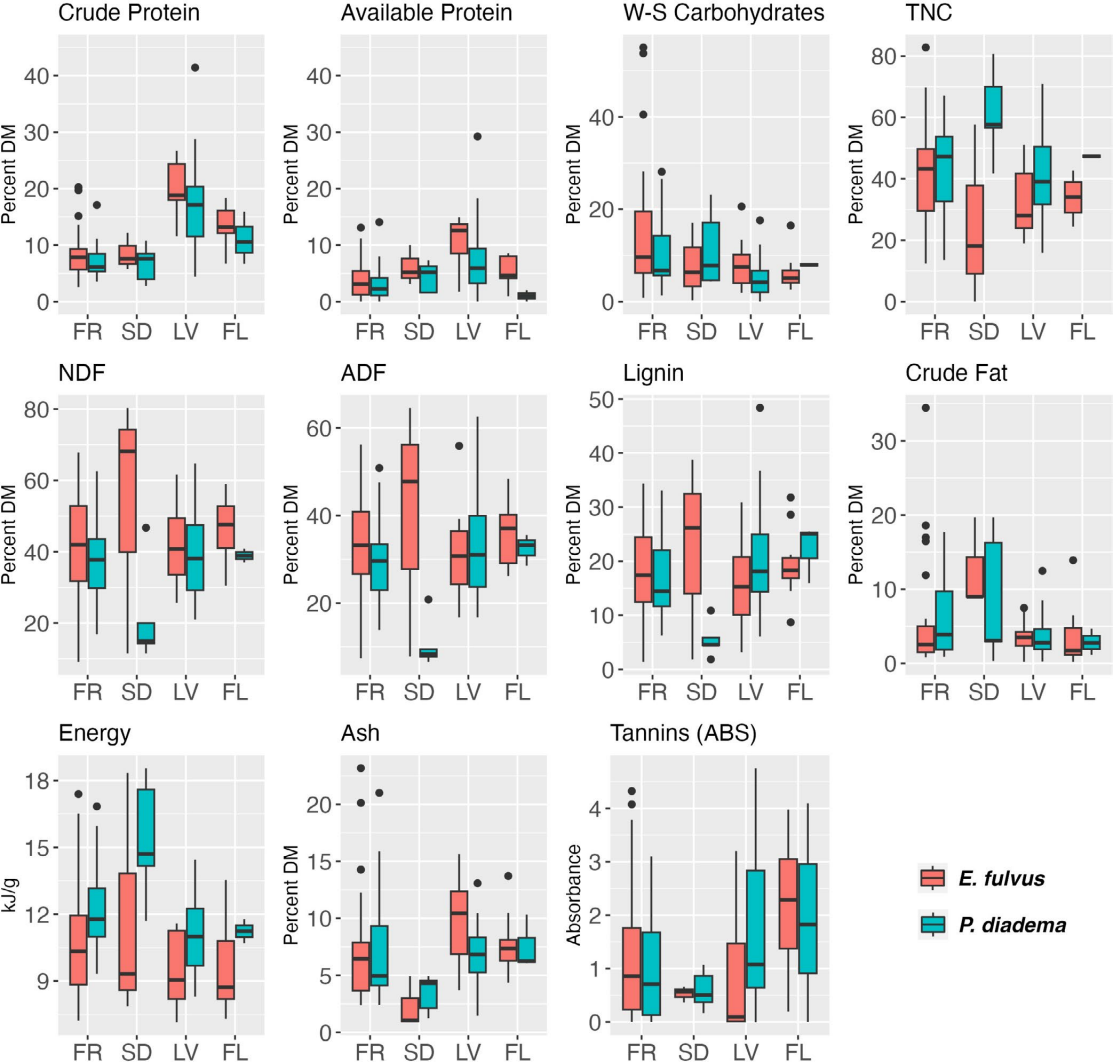
Macronutrient composition of foods of *Eulemur fulvus* and *Propithecus diadema* at Ankadivory, Tsinjoarivo, based on 124 samples; plots include all foods representing > 0.1% of feeding time, except a single exudate consumed by *E. fulvus* . Sample size per plant part ( *E. fulvus* , *P. diadema* ): Fruit (FR: 40, 19, of which 7 were shared) seed (SD: 3, 5, of which 1 was shared), leaves (LV: 11, 31, of which 2 were shared), flower buds and flowers (FL: 11, 3).

**FIGURE 3:**
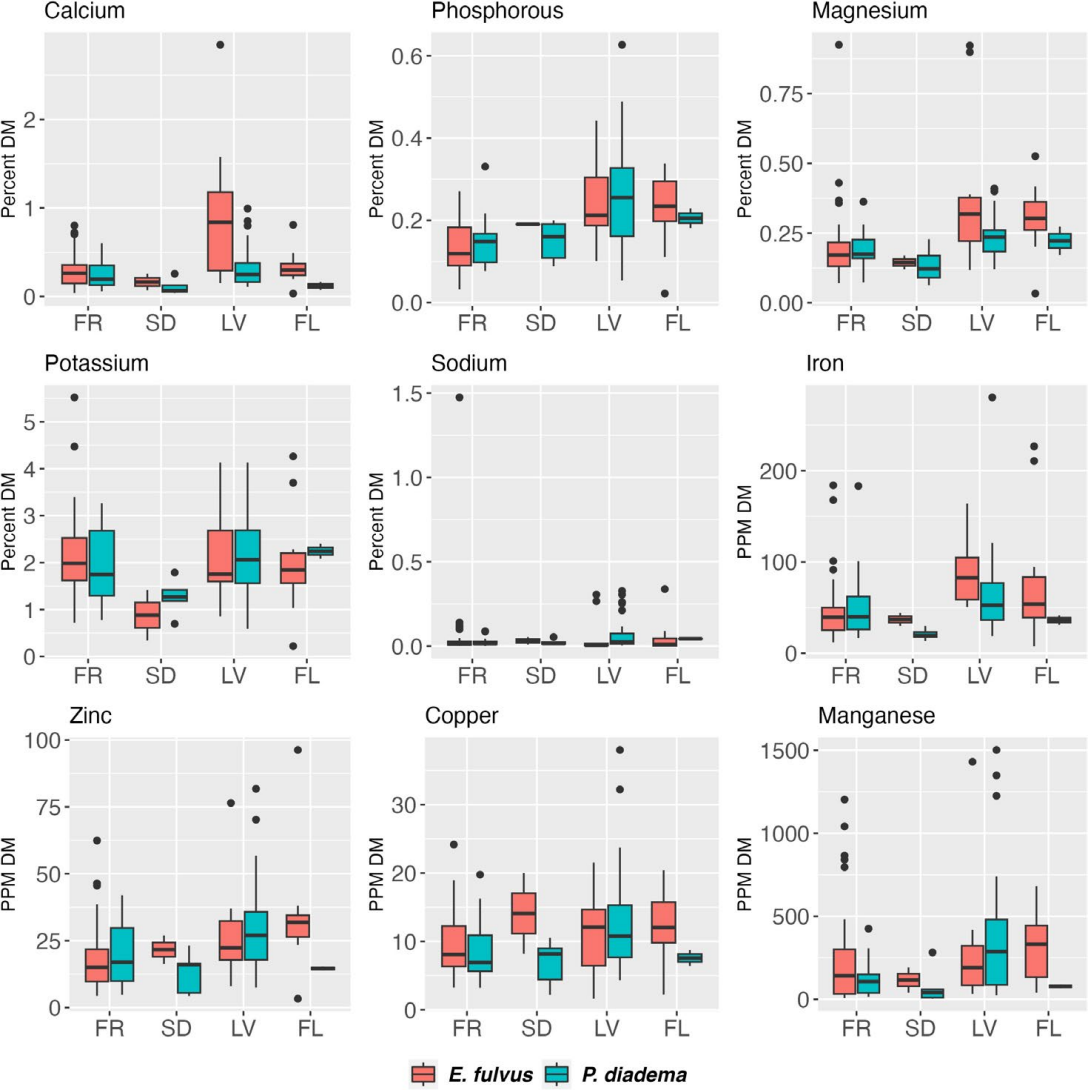
Mineral composition of foods of *Eulemur fulvus* and *Propithecus diadema* at Ankadivory, Tsinjoarivo, based on a total 124 samples; plots include all foods representing > 0.1% of feeding time, except a single exudate consumed by *E. fulvus* . Sample size per plant part ( *E. fulvus* , *P. diadema* ): Fruit (FR: 40, 19, of which 7 were shared) seed (SD: 3, 5, of which 1 was shared), leaves (LV: 11, 31, of which 2 were shared), flower buds and flowers (FL: 11, 3).

As expected, leaves were higher in protein than other foods (Figure 2). Many fruits and seeds were high in fat, but leaves and flowers were low. Fruit and leaves were similar in fiber content. Seeds consumed by the two species differed: *E. fulvus* seeds were high in fiber while *P. diadema* seeds were high in TNC, but the sample size was low. Leaves tended to be the best mineral sources (Figure 3), except that fruits and flowers were high for some (potassium, zinc, copper, manganese), and seeds were high in copper.

Fruits/seeds consumed by the two species were similar nutritionally (Table 4; Figures S1 and S2). In the 20 univariate comparisons of fruits/seeds ( *E. fulvus* vs. *P. diadema* ), only one was significant: ADF ( *E. fulvus* higher). In the 20 bivariate correlations between nutritional variables and importance in the diet, *E. fulvus* consumption correlated positively with magnesium, and *P. diadema* consumption correlated positively with energy (Figures S3 and S4). Tannins were prevalent in both species' fruit/seeds: 35 of 43 (81%) for *E. fulvus* and 21 of 24 (88%) for *P. diadema* .

**TABLE 4:** Tests for nutritive variables predicting food selection among fruit and seed species, including all fruits/seeds contributing > 0.1% of a species' feeding time ( *E. fulvus:* 43, *P. diadema* : 24; 8 foods shared) and 11 non‐foods. Pairwise tests are Wilcoxon rank sum tests, and correlations between nutritive variables and consumption level (% of leaf feeding time) are Spearman correlations.

Nutrient	*E fulvus* foods vs. *P. diadema* foods	Correlation between nutrient and consumption level	
		*E. fulvus*	*P. diadema*
Crude Protein	—	−0.14	−0.02
Available Protein	—	−0.20	0.17
W‐S Carbohydrates	—	−0.20	0.15
TNC	—	−0.20	0.21
NDF	—	0.18	−0.23
ADF	Ef>Pd W = 686, *p* = 0.027	0.10	−0.34
Lignin	—	−0.06	−0.18
Fat	—	−0.02	−0.01
Energy (kJ/g)	n/a^a^	−0.22	0.41*
Ash	—	0.16	−0.33
Tannins (ABS)	—	−0.05	−0.12
Calcium	—	0.13	−0.28
Phosphorous	—	−0.12	0.05
Magnesium	—	0.36*	−0.01
Potassium	—	0.02	−0.19
Sodium	—	0.18	−0.08
Iron	—	0.18	−0.29
Zinc	—	−0.16	−0.30
Copper	—	0.09	−0.01
Manganese	—	0.20	−0.27

*Note:* Because each column has 20 tests and alpha was set at 0.05, 1 significant result per column is expected by chance alone.

Abbreviations: Ef, *Eulemur fulvus* ; F, food; NF, non‐food; Pd, *Propithecus diadema.*

* Denotes significant relationship.

^a^ Not tested because different NDF digestibility coefficients were applied to the two species' energy calculations.

Leaves consumed by the two species showed more divergence (Table 5; Figures S5 and S6). *E. fulvus* leaves were higher in available protein than non‐foods, and their consumption level was correlated with available protein (Figures S7 and S8). There was also noticeable divergence in this variable for the most‐consumed leaves: in the top 5 *E. fulvus* leaves, available protein was consistently high (mean: 11.9%, range 7.1%–14.9%). For *P. diadema* , their top species was high‐protein (18.3%), but the top five averaged 5.6% (range 0%–18.3%). *E. fulvus* leaves were higher in ash than *P. diadema* leaves and non‐foods, suggesting higher mineral content. Neither species selected higher‐energy leaves compared to non‐foods. Tannins were present in 9 of 15 *E. fulvus* leaves (60%), 25 of 31 *P. diadema* leaves (81%), and 8 of 11 non‐foods (73%). *E. fulvus* leaves were higher in calcium and iron than *P. diadema* leaves and non‐foods, and *P. diadema* foods were lower in calcium than non‐foods. *P. diadema* leaves were higher in sodium than *E. fulvus* leaves, and *E. fulvus* leaves were lower than non‐foods. Finally, magnesium concentration correlated with consumption for *P. diadema* .

**TABLE 5:** Tests for nutritive variables predicting food selection among leaf species, including all leaves contributing > 0.2% of a species' leaf feeding time ( *E. fulvus* 16, of which 15 were analyzed; *P. diadema* : 38, of which 31 were analyzed; 6 species shared) and 11 non‐foods. Pairwise tests are Wilcoxon rank sum tests, and correlations between nutritive variables and consumption level (% of leaf feeding time) are Spearman correlations.

Nutrient	*E fulvus* foods vs. *P. diadema* foods	Foods vs. Non‐foods		Correlation between nutrient and consumption level	
		*E. fulvus 15 v. 36*	*P. diadema 31 v. 20*	*E. fulvus*	*P. diadema*
Crude protein	—	—	—	0.38	−0.21
Available protein	—	*F*>NF W = 367, *p* = 0.046	—	0.54*	−0.22
W‐S carbohydrates	—	—	—	0.44	0.09
TNC	—	—	—	−0.15	−0.01
NDF	—	—	—	−0.21	0.13
ADF	—	—	—	−0.35	0.18
Lignin	—	—	—	−0.22	0.18
Fat	—	—		0.19	0.02
Energy (kJ/g)	n/a^a^	—	—	0.10	−0.10
Ash	Ef>Pd *W* = 332, *p* = 0.02	*F*>NF *W* = 413, *p* = 0.003	—	0.30	0.14
Tannins (ABS)	—	—	—	−0.20	−0.22
Calcium	Ef>Pd *W* = 336, *p* = 0.016	*F*>NF *W* = 376, *p* = 0.029	*F*<NF *W* = 164, *p* = 0.005	0.52	0.23
Phosphorous	—	—	—	−0.19	−0.23
Magnesium	—	—	—	0.34	0.42*
Potassium	—	—	—	−0.46	0.26
Sodium	Pd>Ef *W* = 142.5, *p* = 0.036	*F*<NF *W* = 165, *p* = 0.03	—	−0.35	−0.11
Iron	Ef>Pd *W* = 323, *p* = 0.035	*F*>NF W = 401, *p* = 0.007	—	0.03	−0.17
Zinc	—	—	—	−0.13	−0.10
Copper	—	—	—	−0.04	−0.14
Manganese	—	—	—	−0.14	−0.18

*Note:* Because each column has 20 tests and alpha was set at 0.05, 1 significant result per column is expected by chance alone.

Abbreviations: Ef, *Eulemur fulvus* ; F, food; NF, non‐food; Pd, *Propithecus diadema* .

* Denotes significant relationship.

^a^ Not tested because different NDF digestibility coefficients were applied to the two species' energy calculations.

### 3.4 Proportions‐Based Nutritional Geometry

**FIGURE 4:**
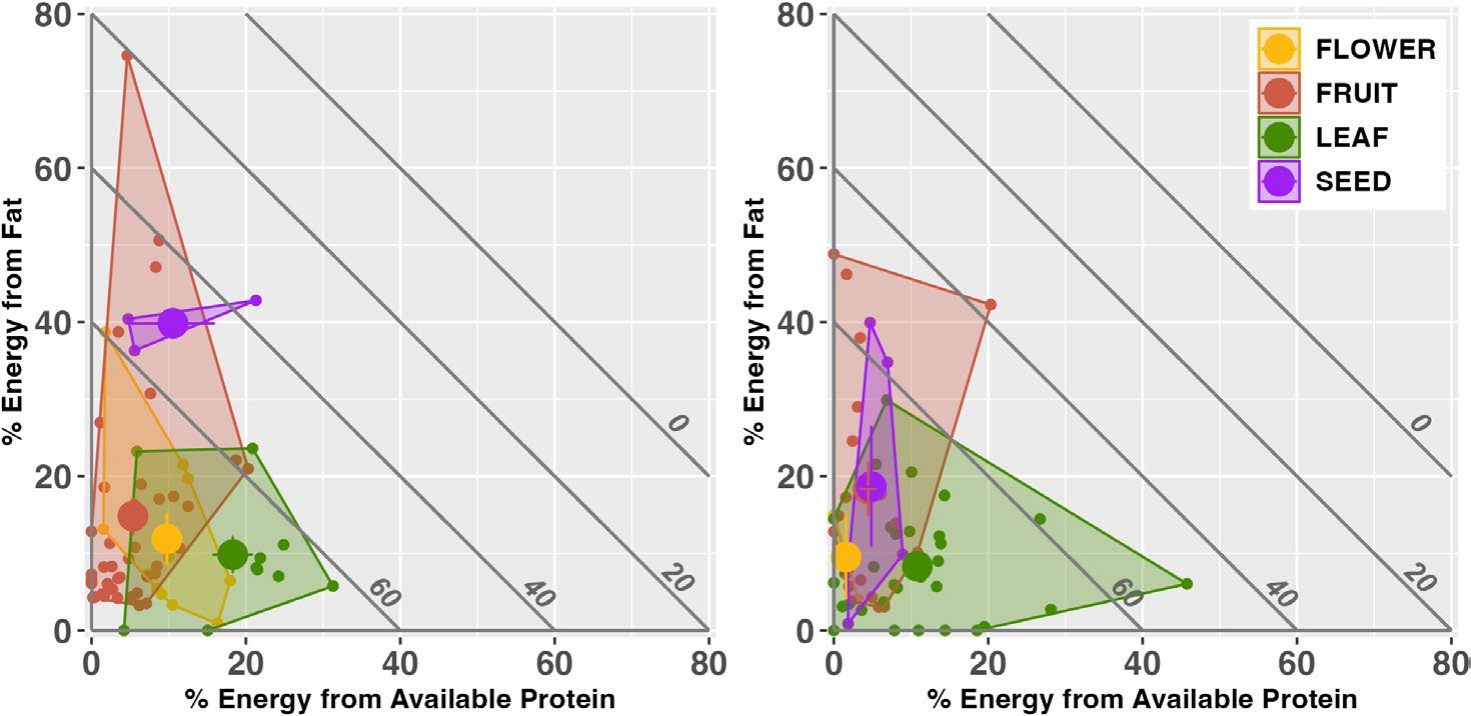
Right‐angled mixture triangle plots showing the relative contribution of protein (*x*‐axis), fat (*y*‐axis) and carbohydrates (TNC plus fiber; implicit z‐axis) to the energy content in food for *Eulemur fulvus* and *Propithecus diadema* at Ankadivory, Tsinjoarivo. The plot displays 123 samples; the sample includes all foods representing > 0.1% of feeding time, except for a single exudate consumed by *E. fulvus* ; labeled diagonal isolines represent the relative contribution of carbohydrates (*z*‐axis); centroids represent mean values.

Right‐angled mixture triangles showing the balance of energy derived from the three macronutrients (Figure 4) revealed broad similarities between the two species in the relative position of food types: leaves showed a higher contribution of protein (lower and to the right) while fruits showed a higher contribution of fat (higher and to the left). There was considerable overlap among food types and between the lemurs for each food type, although the leaf, seed, and flower centroids for *E. fulvus* plotted farther to the right, and their most‐consumed leaf was the most protein‐rich (*Acanthopale madagascariensis*: 14.60% available protein; 31.4% of calories from protein). *E. fulvus* leaves showed a substantially higher contribution from protein; (18.3% vs. 10.8%). Finally, the few seeds targeted by *E. fulvus* were higher in fat than *P. diadema* seeds (39.8% vs. 18.7%).

**FIGURE 5:**
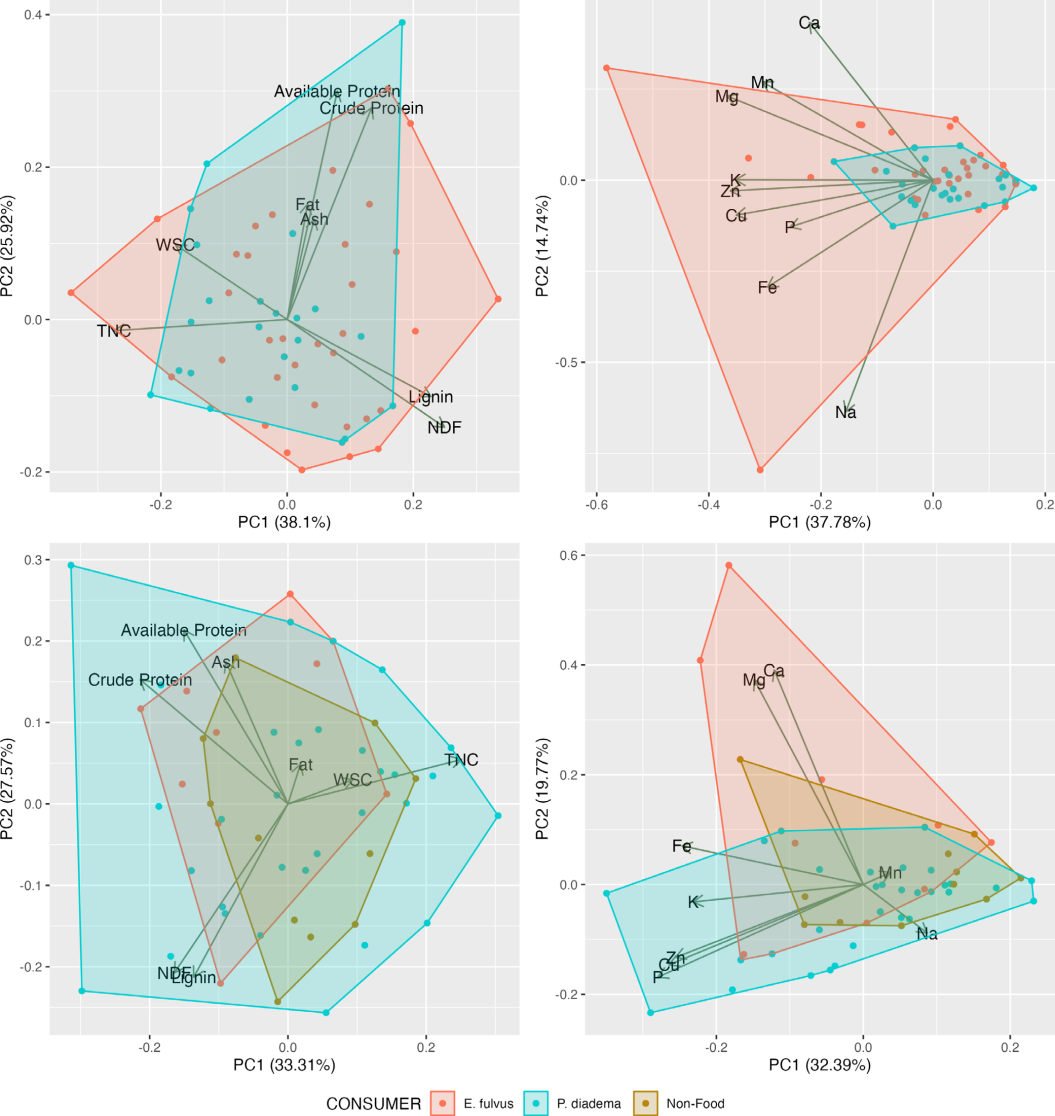
Principal components analysis of fruit/seed foods (top row) and leaves (bottom row) for macronutrients (left) and minerals (right); ADF was removed because of a high correlation with NDF (*r* = 0.90 for leaves, *r* = 0.95 for fruit/seed).

The principal components analysis (Figure 5) successfully reduced the variation to fewer axes (52%–64% of the variation was captured in the first two principal components). Still, for macronutrients, there was little separation among the two lemur species for either fruit/seeds or leaves (and no separation from non‐foods). For minerals, there was substantial overlap between the lemurs, but a greater expansion of the space occupied by *E. fulvus* , representing its tendency to consume higher‐mineral foods (especially Ca and Mg in leaves). The two leaves that drove the positive expansion on PC2 for *E. fulvus* are heavily consumed: *Acanthopale madagascariensis* (topmost point, 20.51% of leaf feeding) and *Isoglossa gracillima* (4.30%), while the two leaf species driving the leftward expansion for *P. diadema* are minor foods: *Polyscias* sp. 3 “Maniny” (0.48% of leaf feeding) and *Albizia gummifera* (0.47%).

## 4 Discussion

### 4.1 Diet and Seasonality

This study confirmed that the labels frugivore and folivore apply to the two study species, in describing the majority of the diet (barely so, in the case of the sifaka consuming 52.7% leaves). However, both fruit and leaves contribute a substantial share of each species' diet. The abundant season appears to be consistent across both lemurs in the focus on fruits and seeds (with *P. diadema* having higher seed consumption), but there is variation in the balance between fruit and seed, and in the timing: *P. diadema* already shifted to high folivory in March/April (late abundant season), while *E. fulvus* stayed devoted to fruit. The lean season strategy is a shift to leaves and/or flowers and varies in two ways. First, both species vary their devotion to leaves and flowers depending on the month; flowers were as high as 30% for *E. fulvus* (September 2016) or 41% for *P. diadema* (July 2016) or as low as < 1%. Second, *P. diadema* 's lean seasons can sometimes include substantial frugivory; June–July 2017 saw a large crop of *Syzygium parkeri* fruits—this species fruits supra‐annually and is unusual in being a lean season fruiter. The willingness of *P. diadema* to exploit this unusual resource aligns with previous studies showing that it prefers fruit/seeds to leaves when available (Irwin 2008). Overall, it appears this variation is driven by what specific resources are available in the lean season, which varies among months and years.

Why *E. fulvus* maintains its devotion to fruit in lean season months when *P. diadema* turns to other food types remains unclear. Activity budgets (Rahalinarivo et al. 2023) showed that these two groups diverged in their lean season strategy: *E. fulvus* was an “energy maximizer”, increasing overall activity and feeding time; this may be related to the scarcity of fruit and the need for more search effort, or the smaller size of the fruits available. Presumably, higher reliance on leaves would not work, as they lack the necessary dental and gastrointestinal adaptations to folivory. *P. diadema* was a “time minimizer”, reducing all activity (feeding, travel and social) while shifting heavily to leaves/flowers. Presumably, for *P. diadema* , doubling down on finding fruits may not yield a higher payoff than conserving energy and relying on leaves (to which they are well‐adapted), and this plasticity also allows exploitation of foods that tend to be more readily available in the animal's immediate vicinity.

*P. diadema* exhibited more flexibility, in the sense that leaves were not its primary food in all months (dipping as low as 24%), while *E. fulvus* fruit consumption never dipped below 56%. This aligns with the findings of Sato et al. (2016); despite the more prominent adaptations to folivory in *Propithecus* species, they exhibit more dietary flexibility than *Eulemur*. Among lemurs, some genera are more dedicated folivores than *Propithecus*, such as the exclusively folivorous *Avahi meridionalis* (Campera et al. 2021). However, even *Varecia*, often described as the lemur most dedicated to frugivory, experiences months when fruits are the minority of the diet (Beeby and Baden 2021), reinforcing previous suggestions that Madagascar's frugivores are limited from complete dedication due to low protein content in Madagascar fruit (Donati et al. 2017).

### 4.2 Food Selection Choices and Nutrient Content

In terms of species consumed, despite considerable overlap in the two species' food lists, there was very little meaningful overlap. Most “shared species” were either consumed for different parts or were exceedingly minor foods for one or both species. This suggests that, despite considerable overlap in broad food categories (fruit, leaves, flowers), the species operate under differing food selection rules (i.e., how choice relates to food chemistry). Our prediction that *P. diadema* , the folivore, would have higher dietary diversity was not upheld, and there was no consistent seasonal difference. More research is needed to unravel the causes and consequences of this variation, especially given that higher dietary diversity can indicate either a worse or better dietary situation (e.g., either low availability of preferred foods necessitating “branching out” with inferior foods, or a high availability of multiple preferred foods).

Nutritional results confirmed some general patterns: leaves deliver more protein while fruits deliver more non‐structural carbohydrates, and fruits/seeds deliver more energy than leaves (Felton, Felton, Wood, et al. 2009; Irwin et al. 2014; Norconk et al. 2009; Rothman, Dierenfeld, et al. 2006), and leaves deliver more of most minerals than fruits, seeds, and flowers (Irwin et al. 2017). However, fruits and leaves were similar in fiber, contrary to previous findings that fruit tend to be lower in fiber (Lambert and Rothman 2015). The previous finding of extremely low calcium values in *P. diadema* foods was confirmed but contrasted with the high levels in *E. fulvus* leaves, meaning that higher‐calcium options are available in the habitat. Though our techniques did not allow quantification of tannin concentrations, their high prevalence in both species' foods suggests a substantial impact on nutritional outcomes, and the lemurs may have counteradaptations (Espinosa‐Gómez et al. 2018). Most *E. fulvus* leaves had tannins (60% of 15 foods tested), but strikingly, their top species, *Acanthopale madagascariensis*, had both high protein and was tannin‐negative, suggesting a high value for this food.

These overall dietary differences likely translate into broadly different intakes: for example, *P. diadema* 's emphasis on leaves likely translates into higher daily protein intakes. This awaits confirmation in this population using daily intake estimates, but would align with many studies showing higher overall levels of protein and fiber in folivores' foods and higher overall levels of nonstructural carbohydrates in frugivore foods (Rothman et al. 2011; Raubenheimer et al. 2015; Lambert and Rothman 2015; Yamashita 2008; Greene et al. 2024).

Proportions‐based nutritional geometry revealed that the choices the two lemurs made within the two major food types (fruit/seed and leaf) showed broad nutritional overlap, but key elements of divergence. Although many variables showed no divergence, *P. diadema* appears to select high‐energy fruits/seeds, while *E. fulvus* prioritizes protein, calcium, and iron in its leaf selections. These results confirm our predictions: the frugivore is more choosy about leaves, and the folivore is more choosy about fruit. These choices likely make sense in the context of overall nutritional intakes. A frugivore eating mainly fruit (a lower‐protein, lower‐mineral food) can compensate by choosing the highest‐protein and highest‐mineral leaves. A folivore eating mainly leaves (lower energy) can compensate by maximizing the energy in the fruits/seeds it consumes.

One puzzling question that needs further study is why sifakas are not more selective in the nutritional quality of their leaves, which make up the bulk of their diet. The strict separation between the species' lists was striking: it was not just that *P. diadema* was being less choosy (including a wider range of species), it was strictly avoiding the *E. fulvus* choices, including the “highest‐quality” leaves in terms of protein and mineral content. Some of the top *E. fulvus* leaves that *P. diadema* ignored were terrestrial herbaceous plants (the high‐value *Acanthopale madagascariensis*, and *Isoglossa gracillima*), but *P. diadema* often forages on the ground for other resources such as subterranean parasitic flowers, *Impatiens* sp. and *Pteridum* sp. (Irwin et al. 2007, 2017). As the folivore, the expectation is that they should be more completely adapted to leaves, including neutralization of PSMs, and therefore have freer choice than frugivores, yet they selected only some, and their choices seemed apparently inferior to those of *E. fulvus* based on the variables reported here. This happened even when they had a fruit‐heavy diet in abundant season months.

One potential explanation for *P. diadema* 's low‐protein, low‐mineral choices is the overall preponderance of leaves in the diet, causing them to meet those needs without being choosy, and that they may prefer non‐protein calories over excess protein calories. Another possibility is that they may experience more thorough absorption of nutrients than suggested by our analyses. In particular, the “AvailN” technique for quantifying available protein (DeGabriel et al. 2008) is thought to account for tannins reducing protein digestibility but does not account for counteradaptations such as tannin‐binding salivary proteins (Espinosa‐Gómez et al. 2018), so protein intake may exceed that suggested by our “available protein” data. Further research is needed to understand true nutrient absorption in both species as well as how digestibility varies across months and dietary shifts.

The general trend suggests both lemurs are more choosy about their minor foods, using those foods strategically to fill key nutritional gaps, and less choosy about major foods (and the nutrients those foods are good at delivering). *E. fulvus* showed no sign of prioritizing foods high in either WSC or TNC despite the conventional wisdom that frugivores “focus” on rapidly digestible carbohydrates, and *P. diadema* showed no sign of prioritizing protein or fiber, the nutrients they likely get in surplus. This suggests *E. fulvus* populations may be limited by protein while *P. diadema* is not.

### 4.3 Implications and Future Directions

This study revealed a striking combination of considerable overlap in plant parts used, extreme divergence in species chosen (especially for leaves), and modest nutritional divergence of the species' foods in just a few key variables. As the two lemurs occupied the same habitat, are both agile and arboreal, and have access to all resources (including terrestrial plants), understanding which resources they use and which they eschew yields key information about nutritional strategies.

Further research is needed to better understand the motivations behind these differing preferences, especially a better characterization of PSMs (which we did not attempt, beyond basic tannin screening) and quantification of nutrient absorption (digestibility). Further research is also needed in this population and others to go beyond characterizing food chemistry and calculate daily nutritional intakes that can be compared across species and seasons (Conklin‐Brittain et al. 2006; Felton, Felton, Raubenheimer, et al. 2009; Irwin et al. 2015; Rothman et al. 2011; Vogel et al. 2015). These go beyond the crude measure of feeding time and account for mass intakes and how these vary across seasons, populations and species. Previous studies of *P. diadema* at Tsinjoarivo documented a large increase (3–4×) in the mass of food ingested in abundant season months (Irwin et al. 2015). In other words, seasonal shifts in nutrient concentrations need not be mirrored by shifts in intakes of those nutrients if the overall amount ingested varies as well. Finally, it is important to note that the results presented here describe one group per species during 1 year—dietary and behavioral outcomes may differ in other groups, habitats, or years.

A better understanding of the folivore and frugivore niches in Madagascar and elsewhere will promote a better understanding of how primate species, both within and across guilds, coexist in their habitats. This informs our understanding of community assembly in the present and how it arose from past niche divergence. In some cases, this will help us understand evolutionary transitions related to microhabitat use and/or activity period, which might be partially driven by interspecific competition. On a practical level, the degree of competition between species is important for understanding a habitat's carrying capacity for individual species and primate communities and the nature of interspecific interactions. From the ecosystem's point of view, the degree of divergence is also important for understanding the redundancy in terms of ecosystem services such as seed dispersal (Wright et al. 2011). This type of knowledge is critical for managing ecosystems as they are increasingly affected by human impact, including the identification of critical resources, which may not be highly consumed species. One such human impact, climate change, has considerable potential to cause primate population declines through disruption of food supply; this can occur through changes in plant communities over medium‐ and longer‐terms and the disruption of plant reproductive cycles over relatively short time frames (Rothman et al. 2024). For example, *Acanthopale madagascariensis* may be critical for *E. fulvus* , comprising just 2.9% of overall feeding but making a disproportionately large contribution in protein and minerals, while the lean‐season fruiting *Syzygium parkeri* may be a key food for *P. diadema* . This application extends naturally to captive management and the formulation of diets that emulate nutritional intakes in the wild.

## Author Contributions

**Mitchell T. Irwin:** conceptualization (lead), data curation (lead), formal analysis (lead), funding acquisition (lead), investigation (lead), methodology (lead), project administration (lead), visualization (lead), writing – original draft (lead), writing – review and editing (lead). **Vololonirina Rahalinarivo:** data curation (supporting), investigation (supporting), project administration (supporting), writing – review and editing (supporting). **Bruno Ramorasata:** data curation (supporting), investigation (supporting), project administration (equal), writing – review and editing (supporting). **Jean‐Luc Raharison:** investigation (supporting), project administration (equal), writing – review and editing (supporting). **Jean‐Freddy Ranaivoarisoa:** investigation (supporting), project administration (supporting), writing – review and editing (supporting). **Chloé N. M. Gherardi:** data curation (supporting), investigation (supporting), writing – review and editing (supporting). **Jessica M. Rothman:** data curation (supporting), investigation (supporting), methodology (supporting), writing – review and editing (supporting).

## Conflicts of Interest

The authors declare no conflicts of interest.

## Supporting information


Figures S1–S8.

Tables S1–S8

## Data Availability

The data and materials underlying this article are available in the article and in its [Link], [Link].
