# Supplementary material for: More Choosy for Minor Foods: Diet, Seasonality and Food Selection in Sympatric Frugivorous and Folivorous Lemurs
Source: Ecol Evol. 2025 Mar 13;15(3):e71069. doi: 10.1002/ece3.71069 (PMC11904312; doi:10.1002/ece3.71069)

**Supplemental Material to Accompany Irwin et al.**

**More choosy for minor foods: Diet, seasonality and food selection in sympatric frugivorous and folivorous lemurs**

**Figure S1:** Violin plots showing nutritional variables for: (1) fruit/seed species consumed by *Eulemur fulvus* (n=43), and (2) fruit/seed species consumed by *Propithecus diadema* (n=24) at Ankadivory, Tsinjoarivo.


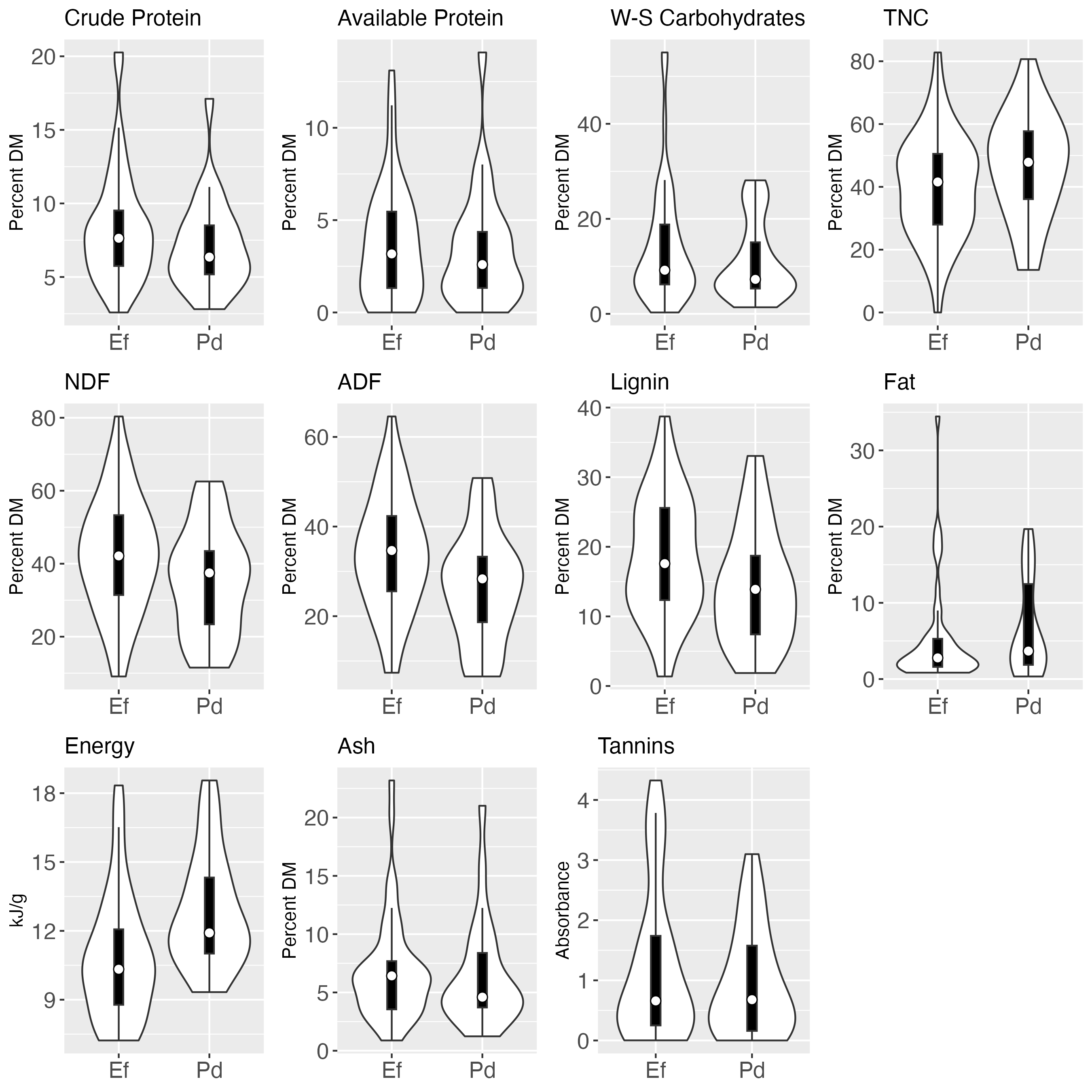


**Figure S2:** Violin plots showing mineral concentrations: (1) fruit/seed species consumed by *Eulemur fulvus* (n=43), and (2) fruit/seed species consumed by *Propithecus diadema* (n=24) at Ankadivory, Tsinjoarivo.


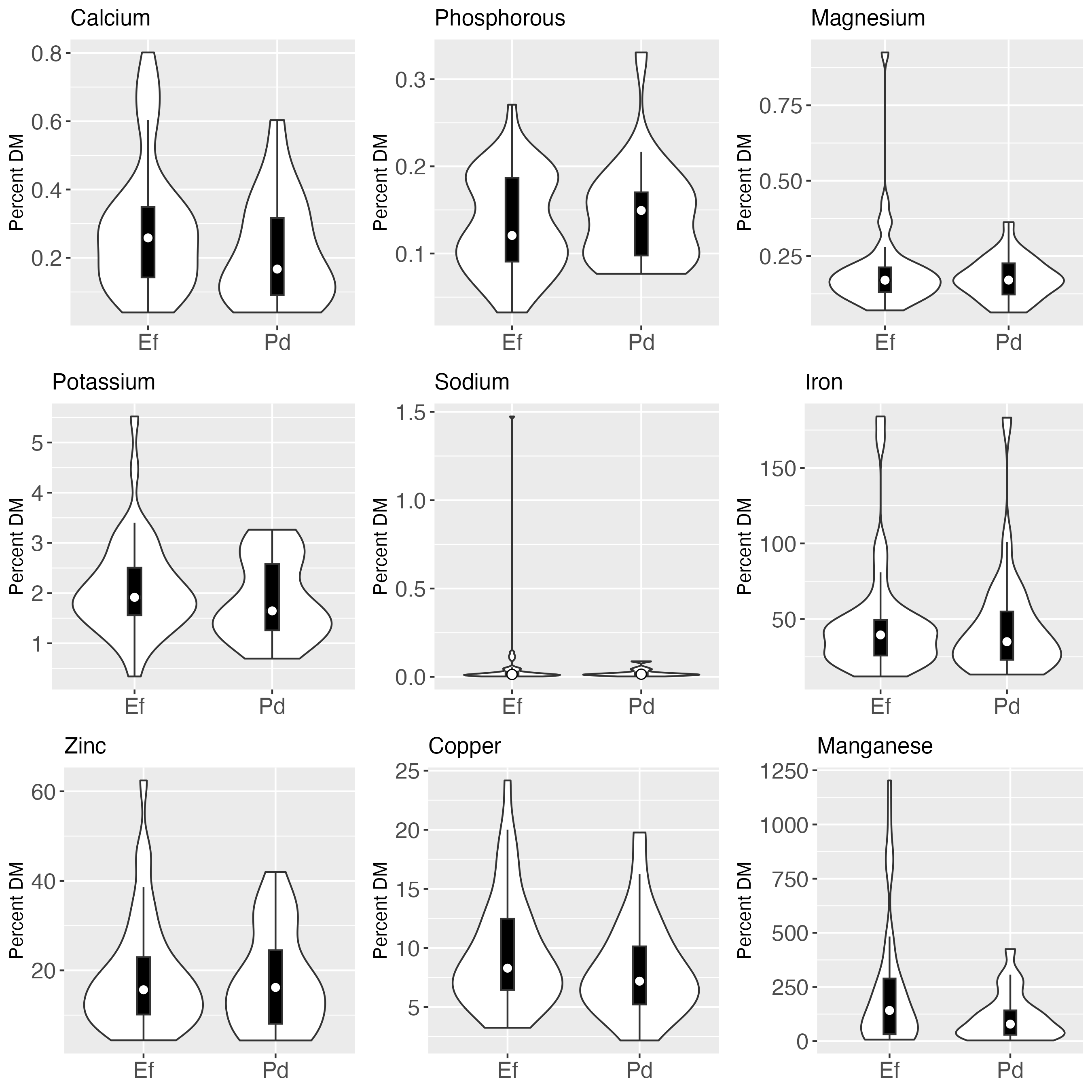


**Figure S3:** Bivariate relationships between food species’ contribution to overall fruit/seed feeding time (y-axis) and nutritional (x-axis) for *Eulemur fulvus* and *Propithecus diadema* at Ankadivory, Tsinjoarivo.


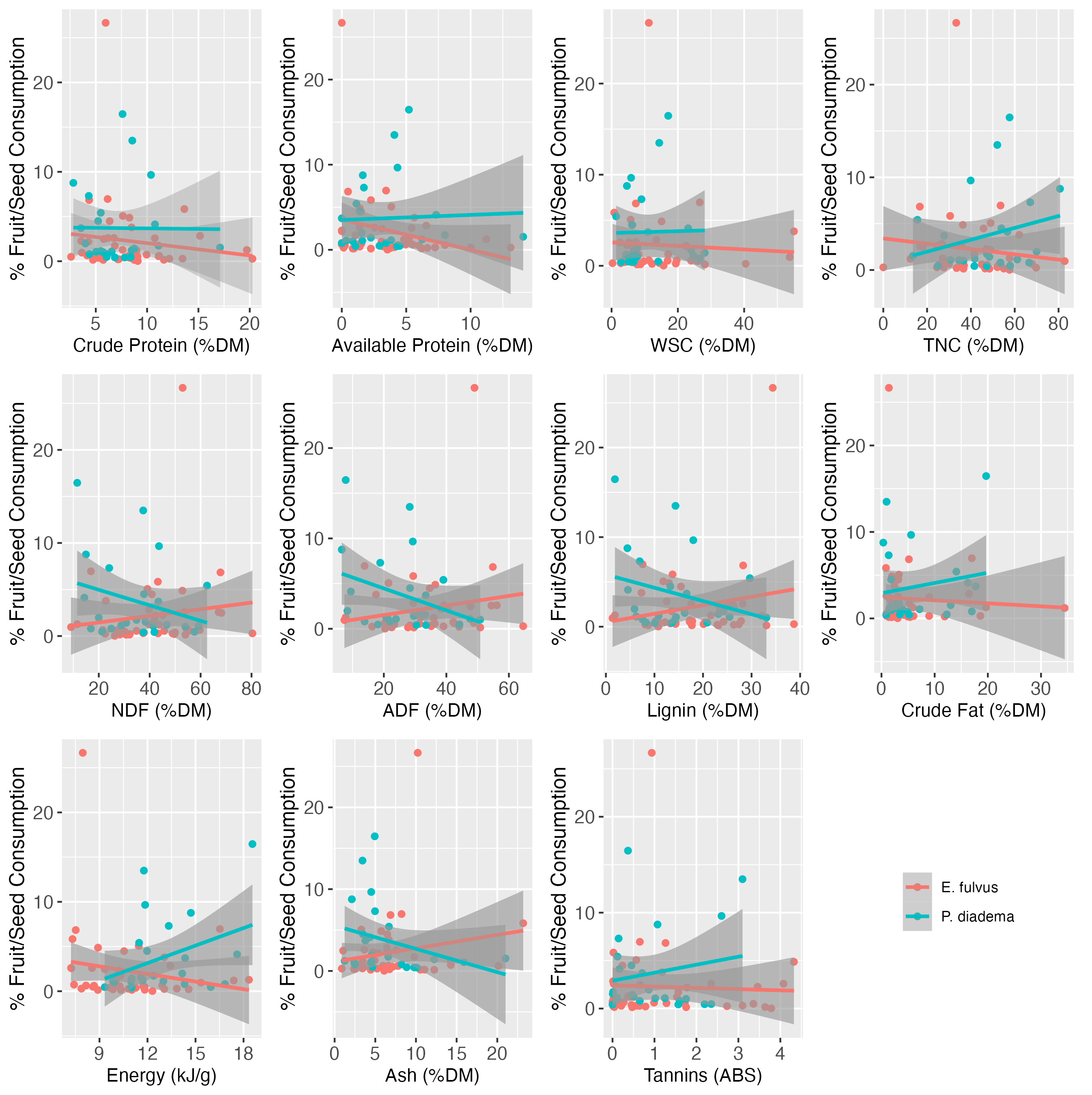


**Figure S4:** Bivariate relationships between food species’ contribution to overall fruit/seed feeding time (y-axis) and mineral concentrations (x-axis) for *Eulemur fulvus* and *Propithecus diadema* at Ankadivory, Tsinjoarivo.


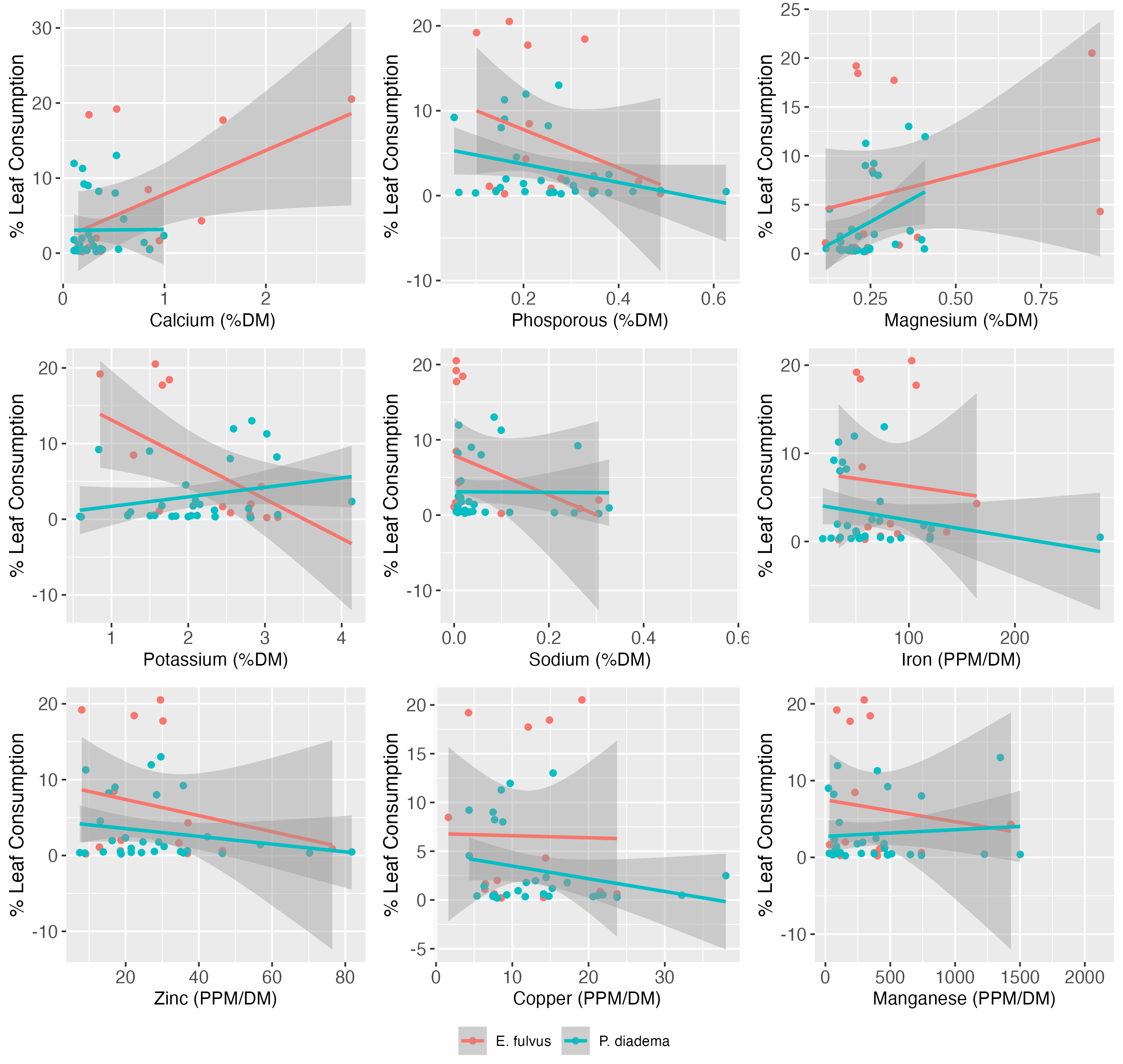


**Figure S5:** Violin plots showing nutritional variables for: (1) leaf species consumed by *Eulemur fulvus* (n=15), (2) leaf species consumed by *Propithecus diadema* (n=31), and (3) leaf species sampled that were consumed by neither study species (n=11) at Ankadivory, Tsinjoarivo; 6 species were on both lemurs’ lists.


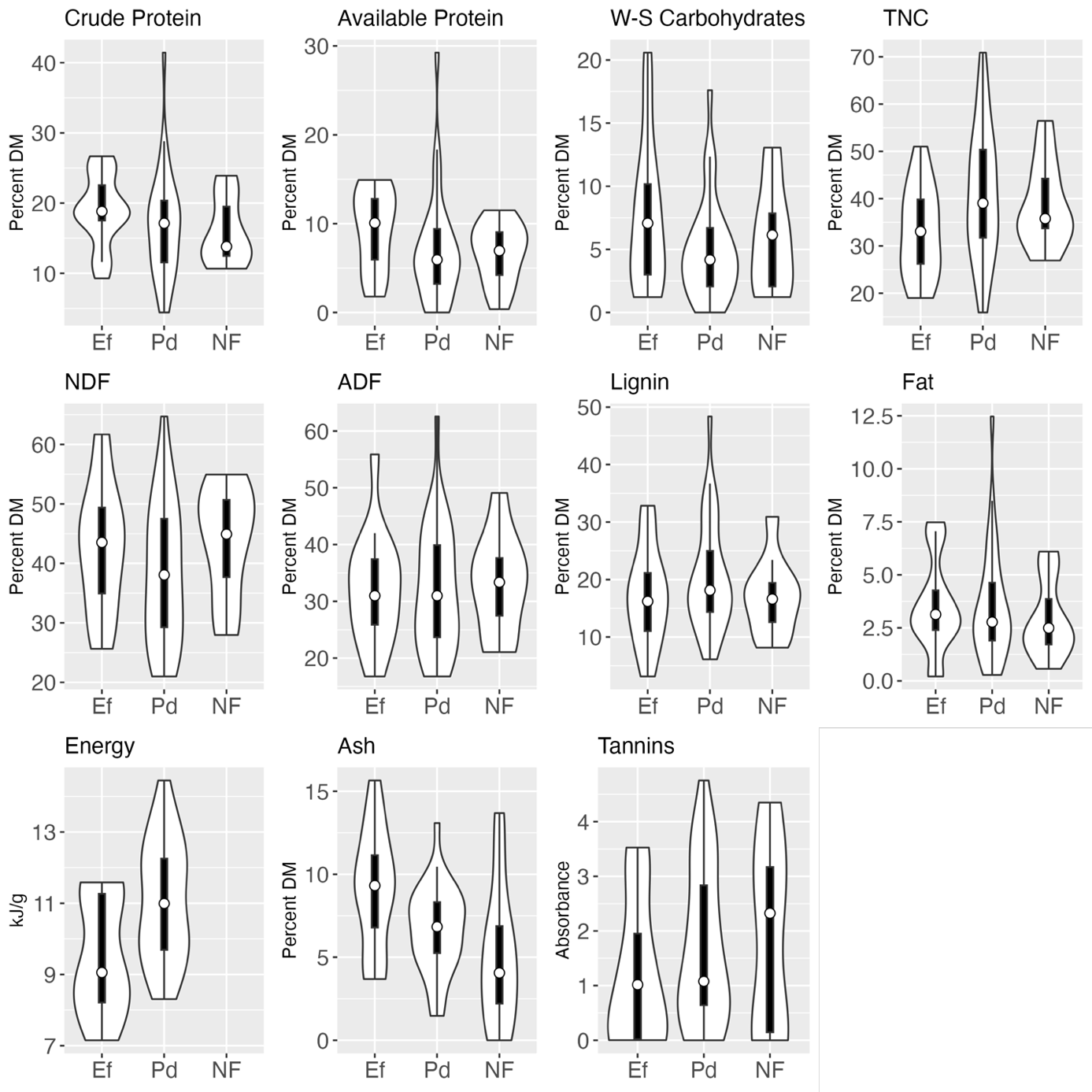


**Figure S6:** Violin plots showing mineral concentrations for: (1) leaf species consumed by *Eulemur fulvus* (n=15), (2) leaf species consumed by *Propithecus diadema* (n=31), and (3) leaf species sampled that were consumed by neither study species (n=11) at Ankadivory, Tsinjoarivo; 6 species were on both lemurs’ lists.


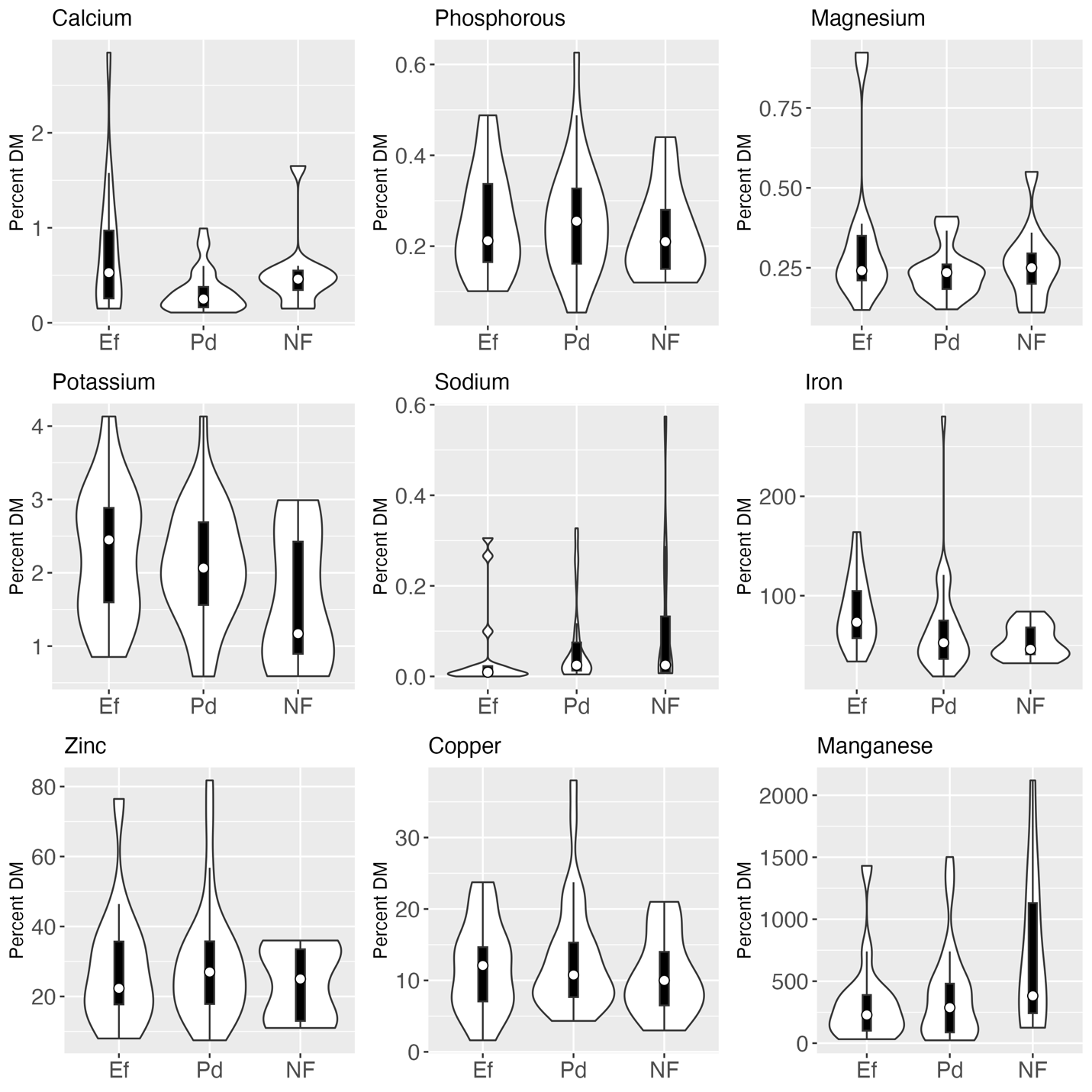


**Figure S7:** Bivariate relationships between food species’ contribution to overall leaf feeding time (y-axis) and nutritional variables (x-axis) for *Eulemur fulvus* and *Propithecus diadema* at Ankadivory, Tsinjoarivo.


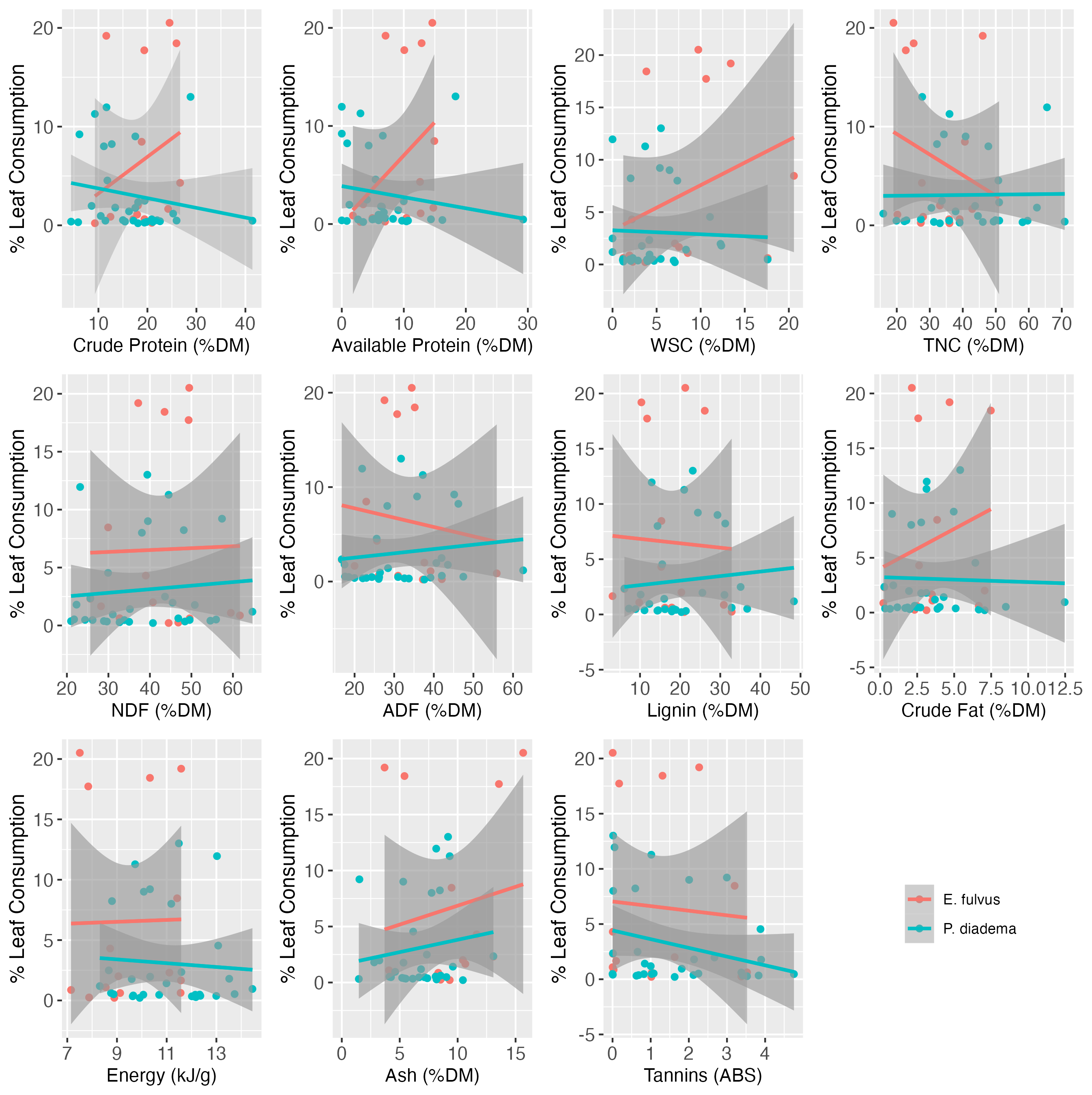


**Figure S8:** Bivariate relationships between food species’ contribution to overall leaf feeding time (y-axis) and mineral concentrations (x-axis) for *Eulemur fulvus* and *Propithecus diadema* at Ankadivory, Tsinjoarivo.


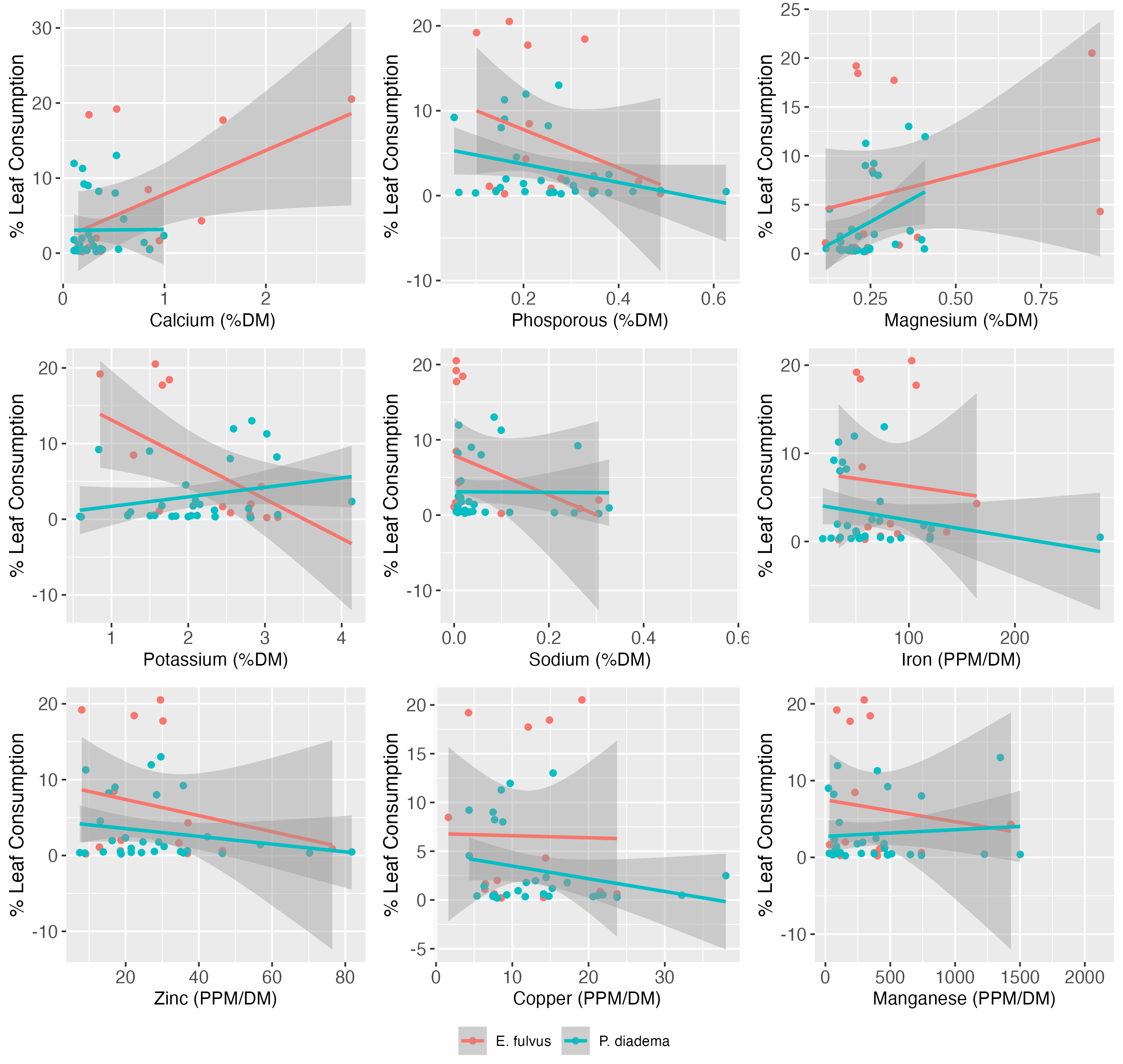

Supplement: Supplementary file 1 — Figures S1–S8. [file ECE3-15-e71069-s001.docx]
